# A logistic paradigm to predict molecular glioblastoma on clinical MRI

**DOI:** 10.3389/fonc.2026.1884886

**Published:** 2026-09-28

**Authors:** Yousif Aldabbagh, Faheem Bhatti, Nitin Menon, Paul S. Morgan, Marion Smits, John A. Maynard, Simon Paine, Robert Goldspring, Stuart Smith, Stefanie C. Thust

**Affiliations:** 1 Precision Imaging, School of Medicine, University of Nottingham, Nottingham, United Kingdom; 2 Radiology Department, Nottingham University Hospitals NHS Trust, Nottingham, United Kingdom; 3 Nottingham NIHRBiomedical Research Centre, Nottingham, United Kingdom; 4 Medical Physics & Clinical Engineering, Nottingham University Hospitals, Nottingham, United Kingdom; 5 Department of Radiology and Nuclear Medicine, Erasmus University Medical Center (Erasmus MC), Rotterdam, Netherlands; 6 Medical Delta, Delft, Netherlands; 7 Imaging Department, Guy’s and St. Thomas’ NHS Foundation Trust, London, United Kingdom; 8 Department of Pathology, Nottingham University Hospitals NHS Trust, Nottingham, United Kingdom; 9 Department of Neurosurgery, Nottingham University Hospitals, Nottingham, United Kingdom; 10 Children’s Brain Tumor Research Centre, University of Nottingham, Nottingham, United Kingdom; 11 Department of Translational Neuroscience and Stroke, UCL Institute of Neurology, London, United Kingdom

**Keywords:** brain tumor, glioblastoma, IDH wild type, logistic regression, MRI, prediction model

## Abstract

**Objective:**

This study aims to externally validate a logistic strategy for predicting the integrated molecular diagnosis of glioblastoma (mGBM) as defined by the World Health Organization (WHO) 2021 Classification of Tumors of the Central Nervous System (CNS).

**Methods:**

Ethical approval was obtained for a retrospective study of diffuse gliomas (CNS WHO grades 2–4) with available isocitrate dehydrogenase (IDH) and 1p19q status and whole genome profiling for mGBM, where appropriate. Age and MRI characteristics (apparent diffusion coefficient (ADC), tumor location, contrast enhancement pattern, cyst(s), and calcification) were scored by two observers blinded to the tissue results and to each other’s results. Observer concordance was assessed using intraclass correlation coefficient (ICC) and Cohen’s kappa. Diagnostic performance, comparisons using McNemar and DeLong tests, and model calibration were obtained in SPSS 27 and Python (v3.12).

**Results:**

A total of 108 consecutively enrolled cases were eligible from January to December 2023 (mean age 56.9 ± 15.5 years; range 20–82), comprising 85 mGBM/IDH wild-type (IDH^wt^) and 23 IDH mutant (IDH^mut^) gliomas. Model sensitivity ranged from 97% to 99%, with 48%–91% specificity, 87%–97% accuracy, and AUCs of 0.93–0.98. For the trainee, model use produced increases in sensitivity from 87.1% to 97.6% with model A and 96.5% with model B and in accuracy from 84.3% to 87.0% and 91.7%, respectively. No within-observer difference in overall accuracy and no paired AUC comparison remained statistically significant after Holm adjustment. Model B demonstrated more consistent calibration across observers. Among eight mGBM lacking rim enhancement, correct classification by the trainee increased numerically from 12.5% to 100% with model A and 87.5% with model B.

**Conclusion:**

The logistic strategy achieved high sensitivity and discrimination for identifying mGBM according to CNS WHO 2021. Model use produced increases in trainee performance, particularly sensitivity, while Model B demonstrated more consistent calibration across observers. These findings support the further evaluation of the tool, particularly in radiologically atypical mGBM.

## Introduction

Diffuse gliomas are the most common primary malignant brain tumor in adults and are mostly incurable. World Health Organization (CNS WHO) grades 2–3 gliomas often progress gradually and are genetically defined by an isocitrate dehydrogenase mutation (IDH^mut^), with or without chromosome 1p and 19q codeletion (1). Over 70% of diffuse gliomas are glioblastoma (GBM), a lethal neoplasm with median survival under 18 months (2). GBM lacks IDH mutations (wild type, IDH^wt^) and exhibits chromosome 7 gain and chromosome 10 loss (+7/-10), epidermal growth factor receptor (EGFR) amplification, and/or telomerase reverse transcriptase TERT promoter mutations (3–5). This predominantly manifests as advanced (CNS WHO grade 4) disease. Tumors lacking CNS WHO grade 4 histomorphology are classed molecular GBM (mGBM) if they qualify by genetic signature.

These may correspond to an earlier disease stage with potential to improve outcomes through radical treatment (6, 7). However, the turnaround time for tissue molecular testing remains variable (8). Henceforth, “mGBM” will describe molecularly defined GBM *of any microscopic grade* and be used interchangeably with IDH^wt^, where results are compared to older published nomenclature. This simplification acknowledges that IDH^wt^ status only partially describes the molecular signature of GBM and that rarer non-diffuse glioma brain neoplasms exist, which lack IDH mutations but are exempt from this work.

Imaging has become central to predicting glioma genetics. Historically, “grading” of gliomas relied on gadolinium contrast patterns (9), but this has limited the sensitivity for mGBM/IDH^wt^ status prediction (10). Apparent diffusion coefficient (ADC) measurements showed potential for IDH genotyping in diffuse gliomas. However, their measurability in rim-enhancing GBM is less established (11, 12). Few biomarkers appear individually accurate (13) and/or lack interobserver comparisons (14).

We developed two multivariable logistic models to predict glioma genotypes on standard clinical MRI within minutes. An individual probability is hereby derived through the addition of log odds ratios for ADC, age, and visual parameters (15). Model A includes scoring of tumor calcification, whereas model B includes scoring of cysts, with similar accuracy achieved in initial testing (15). In model development, qualitative features were stratified by reproducibility (kappa >0.6 and intraclass correlation coefficient (ICC) >0.80 for the diffusion metric (relative ADC mean, rADC_mean_)).

The models originate from a population of CNS WHO grades 2–3 diffuse gliomas (*n* = 290 training, *n* = 49 validation; University College London Hospitals) focused on IDH genotyping preceding the CNS WHO 2021 definition of mGBM (15). This new study aimed to test model performance for identifying mGBM as defined by CNS WHO 2021 and to compare the logistic paradigm results against a diagnostic best guess of an expert and of a trainee.

## Methods

### Patients

Ethics approval for this retrospective imaging data research was obtained (Health Research Authority, United Kingdom, IRAS 326758). Consecutive patients diagnosed through the Nottingham University Hospitals (NUH) brain tumor service, a Tessa Jowell Centre of Excellence, from January to December 2023 were eligible for the study (dates restricted by an unrelated research study). The inclusion criteria were a proven molecular diagnosis of diffuse glioma with available results for IDH and 1p19q genetics, supplemented by further tests and methylation profiling where appropriate to achieve the integrated CNS WHO 2021 diagnosis, and the availability of pre-treatment MRI (16). The exclusion criteria were non-diffuse glioma tissue diagnoses, missing preoperative MRI sequences, and minimal (defined as axial diameter <10 mm) disease.

### Molecular analysis

All tissue samples were processed as formalin-fixed paraffin-embedded blocks and analyzed at our institution’s neuropathology department using the latest methods consistent with the CNS WHO 2021 classification (1). For IDH (R132H)-negative tumors, multiple-gene Sanger sequencing was used to identify alternative IDH mutations. The 1p/19q codeletion status was determined by either fluorescence *in situ* hybridization (FISH) or a quantitative polymerase-chain-reaction-based copy number assay. Where appropriate, tissue samples were further analyzed using whole-genome methylation array (16).

### MRI acquisition

Preoperative imaging was acquired on multiple different 1.5- and 3-T scanners from within the East Midlands Radiology Consortium (EMRAD), which covers six hospital sites for referrals to NUH. All scans included T1-weighted, T2-weighted, T2-FLAIR, and T1-weighted post-contrast image sequences and diffusion-weighted imaging (DWI). DWI was performed using *b*-values of 0 and 1,000 s/mm^2^ in all cases, with some scanners acquiring an additional *b*-value of 500 s/mm^2^. All ADC maps were generated immediately upon DWI acquisition.

### Morphological assessment

The justification for the imaging feature selection, stratified by combined accuracy together with reproducibility (previously six observers), has been detailed in a prior publication (15). Two neuroradiologists (observer 1 (ST) with 14 years of experience and observer 2 (NM), 4th year resident with neuro-oncology reporting experience) independently assessed each case, blinded to tissue data and each other’s results. For each glioma, the location (by tumor epicenter), contrast enhancement pattern (absent, solid-patchy, or rim enhancing with necrosis), presence of cyst(s), and presence of calcification were assessed. Tumor location was categorized into three groups (previously derived for statistical variable reduction according to IDH^wt^ lobar distributions (15)): frontal or insula, thalamic or brainstem, and any other location (e.g., parietal, temporal, or occipital). In the assessment of enhancement, any evidence of necrosis was assigned as a rim enhancement pattern, regardless of necrosis volume. Cysts were defined as foci approximating CSF signal with absent or minimal rim enhancement. Any intratumoral fluid signals not consistent with this description were deemed necrosis. Calcification was assessed using SWI, T2*, and/or b0 sequences. No CT images were available for review in this study because the anonymized MRI data originated from an unrelated research project without access to CT imaging. Examples of the glioma morphologies are shown in **Figure 1**.

**Figure 1:**
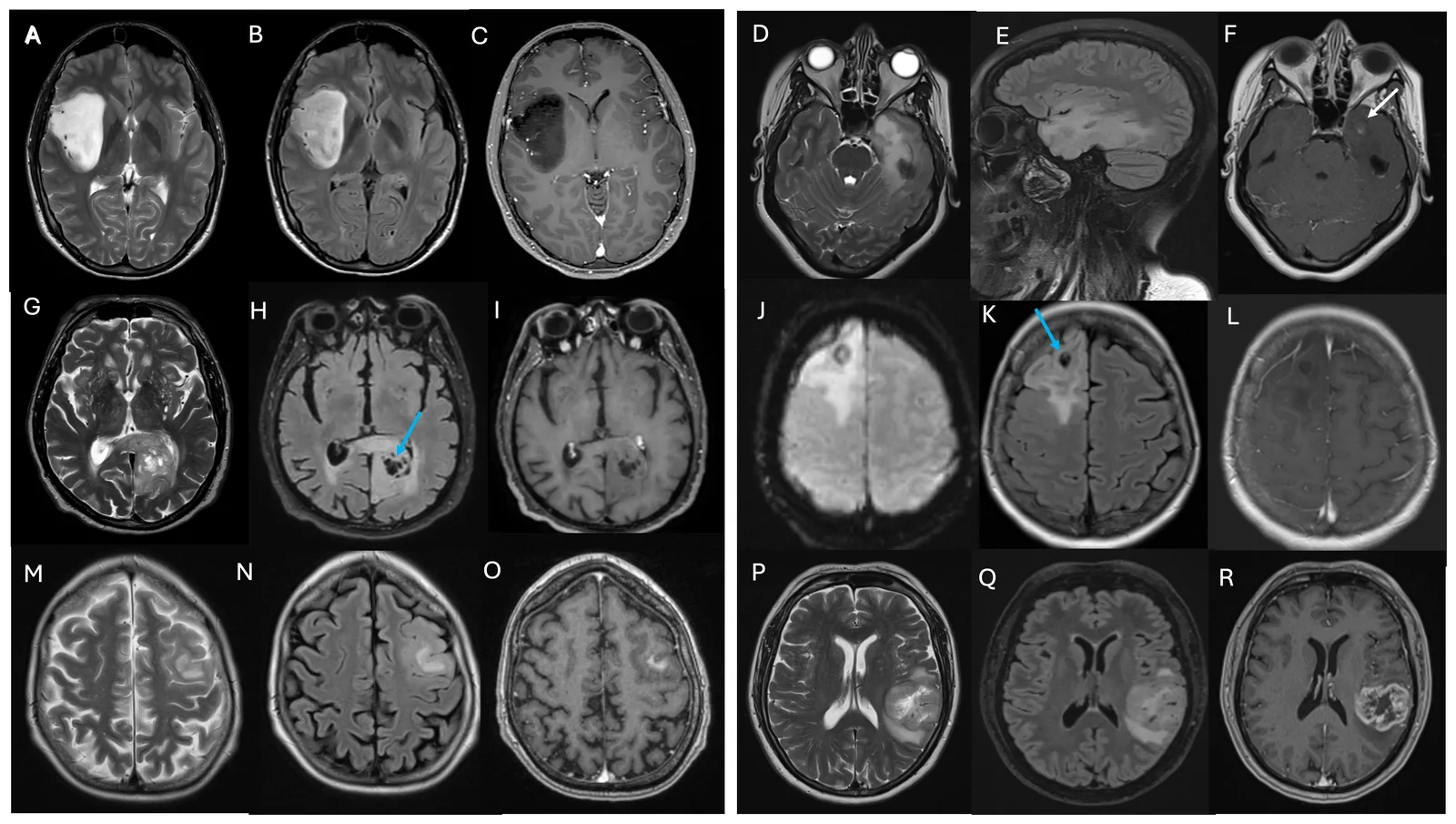
Axial T2-weighted, FLAIR, and post-contrast T1-weighted examples of diffuse glioma morphology: IDH^mut/1p19qintact^ astrocytoma without contrast enhancement (patient 1, **A**–**C**) and with solid-patchy contrast enhancement (patient 2, **D**–**F**, white arrow). Non-contrast-enhancing IDH^mut/1p19codel^ oligodendroglioma with cyst formation (blue arrows) lacking (patient 3, **G**–**I**) and calcification (T2*, **J**) along a cyst wall (patient 4, **J**–**L**). IDH^wt^ glioblastoma (GBM) with solid-patchy enhancement (patient 5, **M**–**O**) and typical GBM manifesting as a rim-enhancing, centrally necrotic mass (patient 6, **P**–**R**). IDH, isocitrate dehydrogenase; mut, mutant; wt, wild type; 1p19q codel, codeletion of chromosome 1 short arm and chromosome 19 long arm.

### ADC quantification

ADC was measured by the same observers (ST, NM) blinded to tissue diagnosis and each other’s results. For non-enhancing and solid-patchy enhancing gliomas, one circular region of interest (ROI) to measure mean ADC (ADC_mean_) was placed in the largest axial tumor cross-section with T2w viewed side by side. In this largest tumor cross-section, the circular ROI ADC_mean_ was chosen as large as possible (but permitting variable size between different tumors) to capture solid tumor while sparing the tumor margin (to minimize partial volume effects in non-spherical lesions). In rim-enhancing tumors, a single circular ADC_mean_ was placed in the largest solid-enhancing tumor focus by viewing contrast-enhanced T1w, T2w, and ADC side by side. In necrotic tumors with thin-rim enhancement, the ADC_mean_ was permitted to capture adjacent non-enhancing tissue, if this was expanded indicative of tumor (**Figure 2**). Macroscopic hemorrhage and calcified elements were excluded from the measurements. A second ROI (ADC_nawm_) was placed in the contralateral centrum semiovale normal-appearing white matter to calculate rADC_mean_ (as the ADC_mean_/ADC_nawm_ ratio). In patients with diffuse infiltration or ischaemic white matter disease, care was taken to spare such areas from ADC_nawm_ measurement.

**Figure 2:**
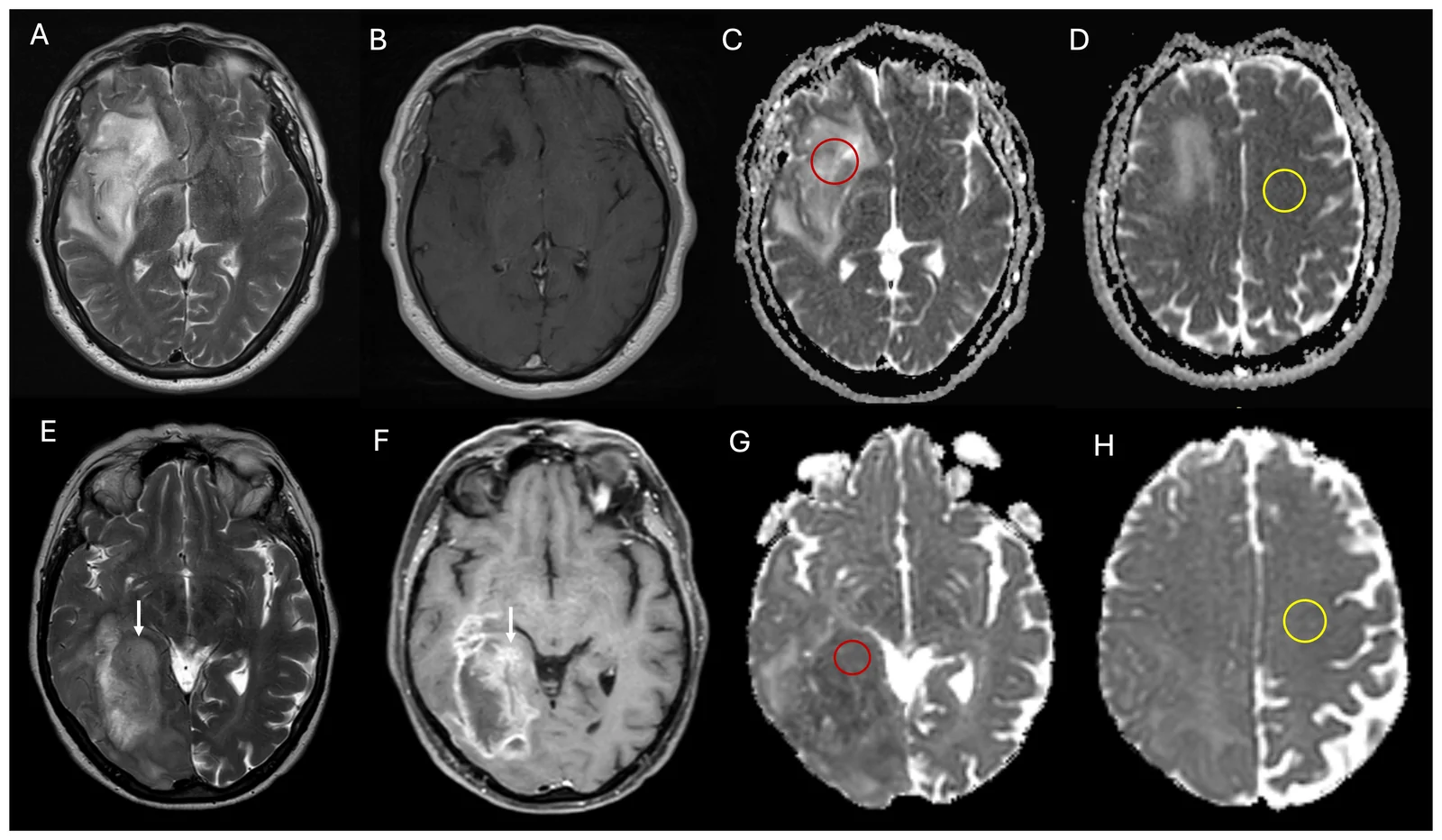
An example of ADC_mean_ (red) and ADC_nawm_ (yellow) measurements with T2-weighted and post-contrast T1-weighted images shown side by side. In macroscopically solid gliomas, one circular ADC_mean_ was measured in the largest axial tumor cross-section, sparing the lesion margin to avoid capture of normal brain parenchyma in non-spherical lesions. In rim-enhancing, centrally necrotic gliomas **(E–H)**, one ADC_mean_ measurement was obtained in the largest area of a solid-appearing tumor defined by expanded tissue within and/or beyond the contrast-enhancing rim (arrows). nawm, normal appearing white matter.

### Logistic modelling of genotype

Through a statistical backward elimination, two multivariable models, A and B, were previously derived to predict IDH genotype. Model A was developed entering all statistically significant univariables, followed by backward elimination. Model B was derived in the same way, but without entry of calcification results to allow for the situation where this information may be unavailable in clinical practice. The logistic regression and dual-model justification are further detailed in (15). The mathematical formula runs in Microsoft Excel, requiring no additional software installation. Here the log odds ratios (LA) for models A and B are calculated (**Table 1**).

**Table 1:** Model A and B formula for the calculation of IDHwt/mGBM probability.

Model A	Model B
- 5.71 × rADC_mean_	-3.23 × rADC_mean_
- 0.05 × age	- 0.095 × age
+ 0.002 × age^2^	+ 0.002 × age^2^
- 0.32 × solid contrast enhancement	- 0.41 × solid contrast enhancement
+ 2.96 × rim contrast enhancement	+ 1.66 × rim contrast enhancement
+ 0.78 × tumor location = other	+ 0.86 × tumor location = other
+ 3.58 × tumor location in thalamus or brainstem	+ 3.64 × tumor location in thalamus or brainstem
+ 4.34 × absent calcification	+ 1.17 × absent cyst
+ 2.24 (constant)	+ 3.07 (constant)
Sum = log odds ratio (L) >> classification by individual probability (mGBM) yes/no)	

The log odds ratios for models A (LA) and B (LB) were hereby calculated as detailed in (15). Model A: [LA = (-5.71 × rADC_mean_) + (-0.05 × age) + (0.002 × age^2^) + (-0.32 × solid contrast enhancement) + (2.96 × rim contrast enhancement) + (0.78 × tumor location = other) + (3.58 × tumor location in thalamus or brainstem) + (4.34 × absent calcification) + 2.24]. Model B: [LB = (-3.23 × rADC_mean_) + (-0.095 × age) + (0.002 × age^2^) + (- 0.41 × solid contrast enhancement) + (1.66 × rim contrast enhancement) + (0.86 × tumor location = other) + (3.64 × tumor location in thalamus or brainstem) + (1.17 × absent cyst + 3.07]. Each tumor is assigned one contrast enhancement category only, which means that a result of 1 is assigned for the tumor’s enhancement category and 0 for the contrast categories that are not applicable. Accordingly, the tumor location is designated 1 for the applicable category and 0 if not in this category. Calcification (model A) and cysts (model B) are coded as 1 if absent and as 0 if present. In the case of an uncertain result of calcification, 1 = absent is entered. The probability of molecular glioblastoma (mGBM) is calculated for models A and B by using the following equation: 1/(1 + *e*^-^*^L^*), where *L* is the relevant log odds ratio. Binary coding was applied with any probability ≥0.5 coded as mGBM, as prespecified in (15).

### Observer prediction of genotype

For each case, the two observers independently provided a subjective prediction of glioma molecular diagnosis, including IDH and 1p19q codeletion status, based on the visual assessment and the knowledge of age.

### Statistical analysis

The statistical analysis and graphs were obtained in SPSS 27 (IBM, Armonk, NY, USA) and Python (v3.12). For each observer and prediction method, sensitivity, specificity, accuracy, positive predictive value (PPV), negative predictive value (NPV), F1 score, and area under the receiver operating characteristic curve (AUC) were calculated with 95% confidence intervals.

Wilson score intervals were used for sensitivity, specificity, accuracy, PPV, and NPV. Two-sided 95% confidence intervals for individual AUC estimates were calculated using the Hanley–McNeil variance method and constrained to the range 0–1. Confidence intervals for F1 scores were obtained from 2,000 non-stratified patient-level bootstrap resamples with replacement.

Patient-paired differences in classification accuracy were assessed using two-sided exact McNemar tests. Paired DeLong tests were used to compare AUCs between systems that generated continuous probabilities. Cross-observer comparisons were matched by patient identifier. Holm adjustment was applied separately to the McNemar and DeLong comparison families, with an adjusted *p*-value <0.05 considered statistically significant. Exact two-sided 95% Clopper–Pearson confidence intervals were calculated for proportions in the small exploratory subgroups.

Calibration was assessed separately for each observer and model using the original continuous predicted probabilities for all 108 patients. Calibration-in-the-large (CITL), calibration intercept, and calibration slope were estimated with model-based Wald 95% confidence intervals. Confidence intervals for the observed-to-expected (O/E) ratio and Brier score were obtained from 2,000 non-stratified patient-level percentile bootstrap resamples with replacement. Logistic recalibration curves were fitted with pointwise 95% bootstrap confidence intervals using the same procedure. No updating of the original model predictions was performed.

The interobserver agreement for ADC measurements was assessed using intraclass correlation coefficient (ICC) analysis with a two-way random-effects model. For the tumor morphological assessment, unweighted and weighted Cohen’s kappa testing was applied. Statistical significance was set at *p* < 0.05.

## Results

From 134 eligible cases, non-diffuse glioma diagnoses (*n* = 12), missing MRI sequences (*n* = 9), duplicates (*n* = 2), MRIs performed after surgery (*n* = 2), and non-measurable disease (*n* = 1) were excluded. A total of 108 patients (mean age 56.9 ± 15.5 years; range 20–82) were included in the final analysis. The study sample consisted of 85 IDH^wt^, 15 IDH^mut^/1p19q^intact^, and eight IDH^mut^/1p19q^codel^ gliomas. The study conforms with the STARD criteria (17). The MRI data selection process is shown in **Figure 3**. An overview of patient demographics and molecular groups is provided in **Table 2**.

**Figure 3:**
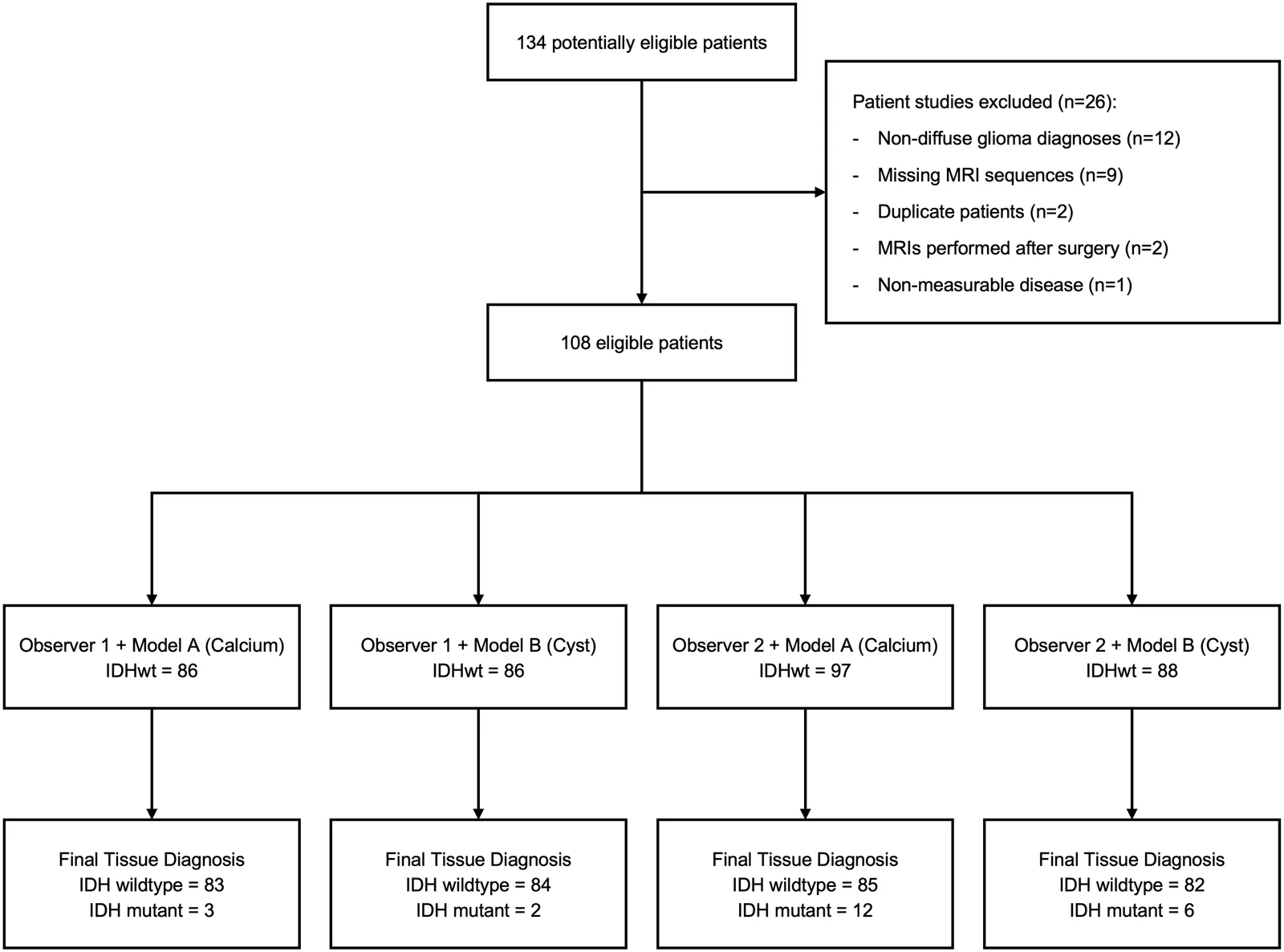
STARD flow diagram of patient selection, modeling results, and final tissue diagnosis. IDH, isocitrate dehydrogenase; mut, mutant; wt, wild type.

**Table 2:** Characterization of glioma molecular subtypes.

Parameter	All gliomas		IDHwt/mGBM		IDHmut/1p19qintact		IDHmut/1p19qcodel	
Number of patients	108 (100%)		85 (78.7%)		15 (13.9%)		8 (7.4%)	
Age in years, mean ± SD (range)	56.86 ± 15.51 (20–82)		61.47 ± 12.63 (20–82)		34.87 ± 8.81 (21–48)		49.13 ± 15.62 (30–79)	
Microscopy CNS WHO grade								
4	87 (80.6%)		83 (97.6%)		4 (26.7%)		0 (0.0%)	
2–3	21 (19.4%)		2 (2.4%)		11 (73.3%)		8 (100.0%)	
Enhancement pattern	Observer 1	Observer 2	Observer 1	Observer 2	Observer 1	Observer 2	Observer 1	Observer 2
Non-enhancing	13 (12.0%)	14 (13.0%)	2 (2.4%)	3 (3.5%)	10 (66.7%)	8 (53.3%)	1 (12.5%)	3 (37.5%)
Patchy or solid enhancing	12 (11.1%)	11 (10.2%)	6 (7.1%)	6 (7.1%)	2 (13.3%)	4 (26.7%)	4 (50.0%)	1 (12.5%)
Rim enhancing	83 (76.9%)	83 (76.9%)	77 (90.6%)	76 (89.4%)	3 (20.0%)	3 (20.0%)	3 (37.5%)	4 (50.0%)
Glioma location	Observer 1	Observer 2	Observer 1	Observer 2	Observer 1	Observer 2	Observer 1	Observer 2
Frontal or insular	44 (40.7%)	43 (39.8%)	28 (32.9%)	27 (31.8%)	9 (60.0%)	9 (60.0%)	7 (87.5%)	7 (87.5%)
Insular	3 (2.8%)	5 (4.6%)	2 (2.4%)	2 (2.4%)	1 (6.7%)	2 (13.3%)	0 (0.0%)	1 (12.5%)
Frontal	41 (38.0%)	38 (35.2%)	26 (30.6%)	25 (29.4%)	8 (53.3%)	7 (46.7%)	7 (87.5%)	6 (75.0%)
Other location	64 (59.3%)	65 (60.2%)	57 (67.1%)	58 (68.2%)	6 (40.0%)	6 (40.0%)	1 (12.5%)	1 (12.5%)
Temporal	31 (28.7%)	31 (28.7%)	27 (31.8%)	27 (31.8%)	4 (26.7%)	4 (26.7%)	0 (0.0%)	0 (0.0%)
Parietal	27 (25.0%)	29 (26.8%)	25 (29.4%)	26 (30.6%)	2 (13.3%)	2 (13.3%)	0 (0.0%)	1 (12.5%)
Occipital	6 (5.6%)	5 (4.6%)	5 (5.9%)	5 (5.9%)	0 (0.0%)	0 (0.0%)	1 (12.5%)	0 (0.0%)
Thalamus or brainstem group	0 (0.0%)	0 (0.0%)	0 (0.0%)	0 (0.0%)	0 (0.0%)	0 (0.0%)	0 (0.0%)	0 (0.0%)
Calcification assessment	Observer 1	Observer 2	Observer 1	Observer 2	Observer 1	Observer 2	Observer 1	Observer 2
Non-calcified	101 (93.5%)	107 (99.1%)	83 (97.6%)	85 (100.0%)	14 (93.3%)	14 (93.3%)	4 (50.0%)	8 (100.0%)
Calcification	7 (6.5%)	1 (0.9%)	2 (2.4%)	0 (0.0%)	1 (6.7%)	1 (6.7%)	4 (50.0%)	0 (0.0%)
Cyst assessment	Observer 1	Observer 2	Observer 1	Observer 2	Observer 1	Observer 2	Observer 1	Observer 2
Non-cystic	97 (89.8%)	98 (90.7%)	84 (98.8%)	83 (97.6%)	9 (60.0%)	10 (66.7%)	4 (50.0%)	5 (62.5%)
Cystic	11 (10.2%)	10 (9.3%)	1 (1.2%)	2 (2.4%)	6 (40.0%)	5 (33.3%)	4 (50.0%)	3 (37.5%)

Details of age, microscopic CNS WHO grade, enhancement category, glioma location (by epicenter), calcification, and cyst rating.

IDH, isocitrate dehydrogenase; mut, mutant; wt, wild type; 1p19q, chromosome 1 short arm and chromosome 19 long arm; mGBM, genetically defined glioblastoma (by molecular analysis).

### ADC quantification

Observer 1 and observer 2 measured average rADC_mean_ values for IDH^wt^ mGBM of 1.3 ± 0.3 and 1.2 ± 0.3, 2.2 ± 0.5, and 2.0 ± 0.7, respectively for IDH^mut^/1p19q^intact^ tumors and 2.0 ± 0.3 versus 1.6 ± 0.2 for IDH^mut^/1p19q^codel^ tumors, respectively. ADC quantification showed good to excellent reproducibility with ICC values of 0.79 for rADC_mean_, 0.80 for ADC_mean_, and 0.97 for ADC_nawm_ (*p* < 0.001 for all). Detailed ICC test results are shown in **Table 3**. The distribution of rADC_mean_ values of the three glioma molecular groups is demonstrated in **Figure 4**.

**Table 3:** Intraclass correlation coefficient (ICC) values categorized by type of ADC region of interest measurement, for all glioma cases.

Variable	ICC measured by	Individual measures ICC (95% CI)	Average measures ICC (95% CI)	Significance	*N*
rADC_mean_	Consistency	0.79 (0.70–0.85)	0.88 (0.82–0.92)	*p* < 0.001	108
	Absolute agreement	0.76 (0.64–0.84)	0.87 (0.78–0.91)	*p* < 0.001	108
ADC_mean_	Consistency	0.80 (0.72–0.86)	0.89 (0.84–0.92)	*p* < 0.001	108
	Absolute agreement	0.77 (0.61–0.85)	0.87 (0.76–0.92)	*p* < 0.001	108
ADC_nawm_	Consistency	0.97 (0.96–0.98)	0.99 (0.98–0.99)	*p* < 0.001	108
	Absolute agreement	0.97 (0.95–0.98)	0.99 (0.98–0.99)	*p* < 0.001	108

ADC, apparent diffusion coefficient.

**Figure 4:**
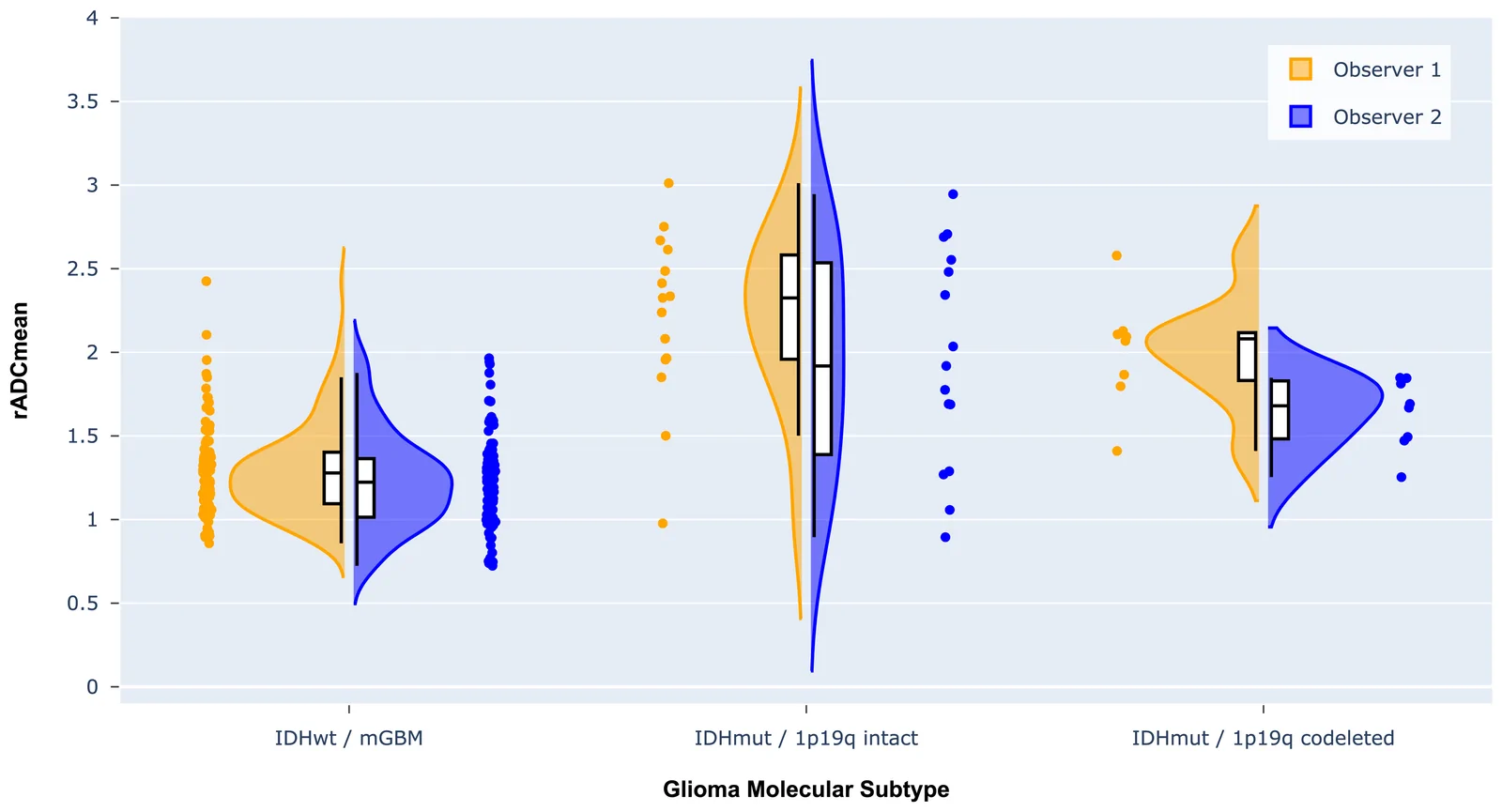
Relative ADC_mean_ (rADC_mean_) distribution by glioma molecular subtype and observer. Boxplots represent the first quartile, median, third quartile, and range of values. rADC_mean_ was derived as the ADC_mean_/ADC_nawm_ ratio. ADC, apparent diffusion coefficient; IDH, isocitrate dehydrogenase; mut, mutant; wt, wild type; 1p19q, chromosome 1 short arm and chromosome 19 long arm.

### Morphological assessment

The weighted kappa agreement between the two observers for tumor enhancement pattern was 0.82 (*p* < 0.001). A total of 83 (76.9%) tumors showed rim enhancement, 12 (11.1%) tumors had patchy or solid enhancement, and 13 (12.0%) gliomas lacked enhancement. The Cohen’s kappa result for tumor location (frontal or insula, thalamic or brainstem, and any other location) was 0.90 (*p* < 0.001). Of the 108 gliomas, 44 (40.7%) were classified as “frontal” or “insula” and 64 (59.3%) as “other”, which means neither frontal, insular, and thalamus nor brainstem. For the assessment of the presence of calcification, Cohen’s kappa between the two observers was -0.016 (*p* = 0.79). Seven (6.5%) tumors were observed to contain calcification. For the assessment of cysts, Cohen’s kappa between the two observers was 0.84 (*p* < 0.001). Cysts were observed in 11 (10.2%) of the tumors. A breakdown of morphological features by molecular group is provided in **Table 2**, and the interobserver analysis for morphological features is listed in **Table 4**.

**Table 4:** Analysis of the interobserver agreement for morphological features using kappa statistics.

Variable	Kappa (95% CI)	*P*-value	Interpretation of kappa (agreement)
Enhancement pattern	0.82 (0.72–0.93)^a^	*p* < 0.001	Almost perfect
Non-enhancing	0.79 (0.61–0.97)	*p* < 0.001	Substantial
Patchy or solid enhancing	0.56 (0.31–0.82)	*p* < 0.001	Moderate
Rim enhancing	0.84 (0.72–0.96)	*p* < 0.001	Almost perfect
Glioma location	0.90 (0.82–0.99)	*p* < 0.001	Almost perfect
Frontal or insula	0.90 (0.82–0.99)	*p* < 0.001	Almost perfect
Other	0.90 (0.82–0.99)	*p* < 0.001	Almost perfect
Thalamus or brainstem	–	*-*	–
Glioma location (lobe)	0.78 (0.69–0.87)	*p* < 0.001	Substantial
Assessment of calcification	-0.02 (-0.05–0.01)	0.79	Poor
Assessment of cysts	0.84 (0.67–1.00)	*p* < 0.001	Almost perfect

^a^ Weighted kappa using linear scale.

### Multivariable model performance

When used by observer 1 (expert), model A correctly classified IDH^wt^ mGBM versus IDH^mut^ status in 103 of 108 gliomas (95.4%) using a likelihood cutoff value of 0.5, with 97.6% sensitivity, 87.0% specificity, and an AUC of 0.98 (95% CI: 0.95–1.00). Model B correctly classified IDH status in 105 of 108 gliomas (97.2%) using the same likelihood cut off value of 0.5. Model B achieved 98.8% sensitivity, 91.3% specificity, and an AUC of 0.96 (95% CI: 0.93–1.00) for identifying IDH^wt^ mGBM.

When used by observer 2 (trainee), model A correctly classified IDH status in 94 of 108 gliomas (87.0%) using a likelihood cutoff value of 0.5, with 97.6% sensitivity, 47.8% specificity, and AUC of 0.93 (95% CI: 0.88–0.98). Model B correctly classified IDH status in 99 of 108 gliomas (91.7%) using a likelihood cutoff value of 0.5, achieving 96.5% sensitivity, 73.9% specificity, and an AUC of 0.94 (95% CI: 0.90–0.98) for identifying IDH^wt^ mGBM. The ROC curves for the two models are shown in **Figure 5**.

**Figure 5:**
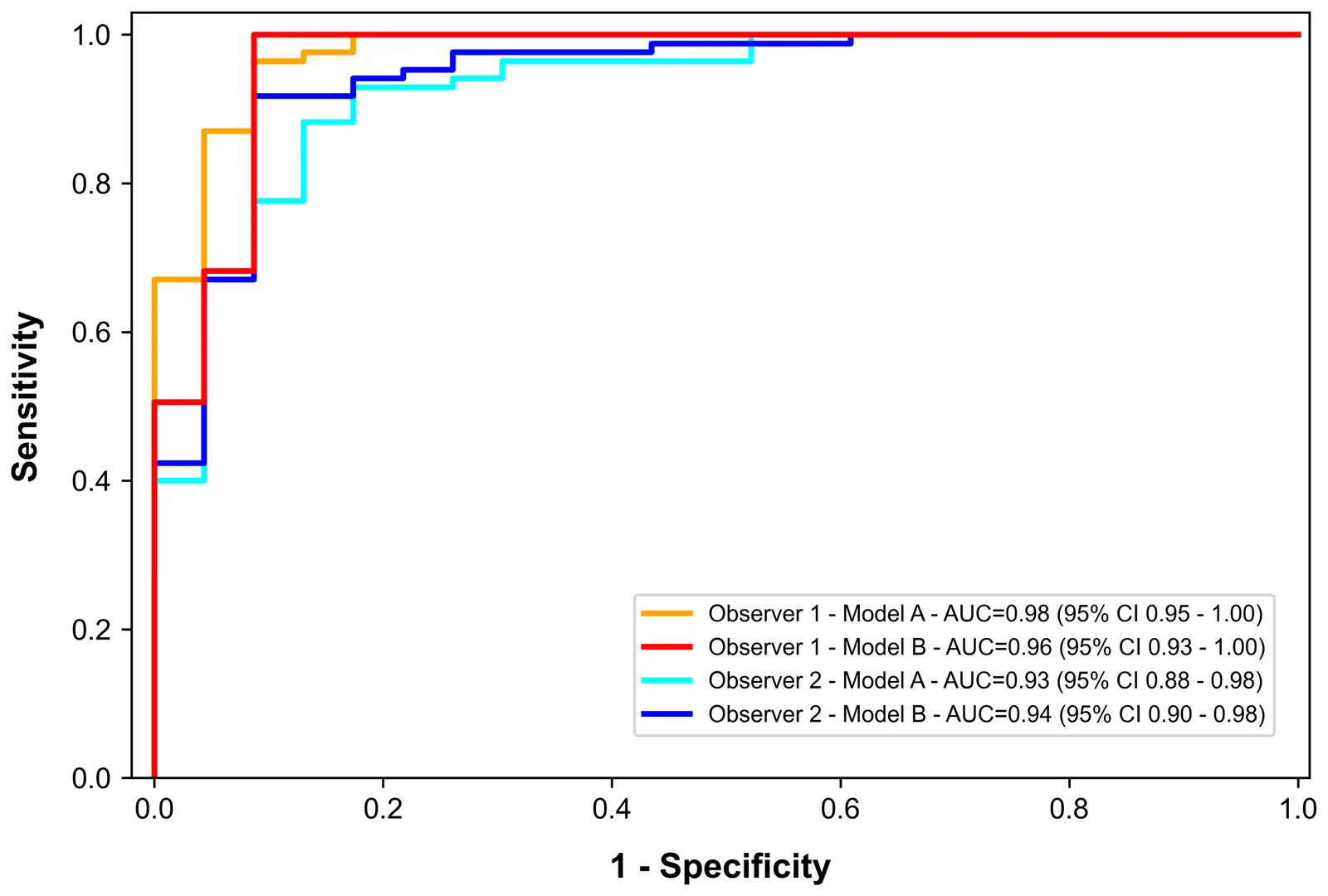
Receiver operating characteristic curves and area under the curve (AUC) values for the prediction of molecular glioblastoma (IDHwt/mGBM) through models A and B when used by observers 1 and 2. Model A assessed for calcification, while model B assessed for cysts.

### Diagnostic performance with and without model use

Complete diagnostic performance results are presented in **Table 5**. Observer 1 (expert) correctly classified 104 gliomas (96.3%), with 98.8% sensitivity, 87.0% specificity, 96.6% PPV, 95.2% NPV, and an F1 score of 0.98. Observer 2 (trainee) correctly classified 91 gliomas (84.3%), with 87.1% sensitivity, 73.9% specificity, 92.5% PPV, 60.7% NPV, and an F1 score of 0.90. For observer 2, accuracy increased to 87.0% with model A and 91.7% with model B, with corresponding F1 scores of 0.92 and 0.95. However, within-observer differences were not significant following Holm adjustment (all adjusted McNemar *p* ≥0.46). Observer 1 performed significantly better than observer 2 when unaided (adjusted *p* = 0.004), but not when using model A or model B (adjusted *p* = 0.12 and *p* = 0.28, respectively). None of the paired comparisons of model AUC remained statistically significant after Holm adjustment (all adjusted DeLong *p* ≥0.10; **Supplementary Table S1**). Complete patient-paired comparisons of classification accuracy using exact McNemar tests are presented in **Supplementary Table S2**.

**Table 5:** Molecular glioblastoma/IDH-wild type was designated the positive outcome.

Model and result	Sensitivity (95% CI)	Specificity (95% CI)	Accuracy (95% CI)	PPV (95% CI)	NPV (95% CI)	F1 score (95% CI)	AUC (95% CI)
Observer 1	0.99 (0.94–1.00)	0.87 (0.68–0.95)	0.96 (0.91–0.99)	0.97 (0.90–0.99)	0.95 (0.77–0.99)	0.98 (0.95–0.99)	–
Observer 1 and model A	0.98 (0.92–0.99)	0.87 (0.68–0.95)	0.95 (0.90–0.98)	0.97 (0.90–0.99)	0.91 (0.72–0.97)	0.97 (0.94–0.99)	0.98 (0.95–1.00)
Observer 1 and model B	0.99 (0.94–1.00)	0.91 (0.73–0.98)	0.97 (0.92–0.99)	0.98 (0.92–0.99)	0.95 (0.78–0.99)	0.98 (0.96–1.00)	0.96 (0.93–1.00)
Observer 2	0.87 (0.78–0.93)	0.74 (0.54–0.87)	0.84 (0.76–0.90)	0.93 (0.85–0.97)	0.61 (0.42–0.76)	0.90 (0.84–0.94)	–
Observer 2 and model A	0.98 (0.92–0.99)	0.48 (0.29–0.67)	0.87 (0.79–0.92)	0.87 (0.79–0.93)	0.85 (0.58–0.96)	0.92 (0.88–0.96)	0.93 (0.88–0.98)
Observer 2 and model B	0.96 (0.90–0.99)	0.74 (0.54–0.87)	0.92 (0.85–0.96)	0.93 (0.86–0.97)	0.85 (0.64–0.95)	0.95 (0.91–0.98)	0.94 (0.90–0.98)

Model A incorporates calcification and Model B incorporates cyst assessment. Model classifications used a probability threshold of ≥0.50. AUC is not presented for unaided observer assessment because this was a binary judgment without a continuous confidence score.

PPV, positive predictive value; NPV, negative predictive value; AUC, area under the receiver operating characteristic curve.

Eight (9.4%) of the mGBM were assessed as non-enhancing or solidly enhancing by observer 1. Of those, all were correctly diagnosed by observer 1 (expert) upon visual assessment, and seven (87.5%) were correctly diagnosed when observer 1 used model A or model B. Only one (12.5%) of the mGBM lacking rim enhancement was correctly diagnosed by observer 2 (trainee) upon visual assessment; however, all 8 (100%, 95% CI: 63.1%–100.0%) mGBM were correctly classified when observer 2 used model A and seven (87.5%, 95% CI; 47.3%–99.7%) when model B was used. The trainee reported a preference for model B, which does not require calcification assessment on MRI.

Two mGBM were microscopically described as CNS WHO grades 2–3. The genotype of these gliomas was correctly identified by observer 1 (expert) through visual assessment alone and when using model A or B. Observer 2 (trainee) misclassified these through visual assessment alone but correctly identified both gliomas when using model A or B.

### Model calibration

Calibration varied by model and observer (**Supplementary Figure S1**; **Supplementary Table S3**). Model B demonstrated the most consistent calibration, with slopes of 1.13 (95% CI: 0.62–1.64) and 0.88 (95% CI: 0.50–1.25), O/E ratios of 1.03 (95% CI: 0.99–1.08) and 1.00 (95% CI: 0.94–1.05), and Brier scores of 0.04 (95% CI: 0.01–0.06) and 0.06 (95% CI: 0.03–0.10) for observers 1 and 2, respectively. Calibration was poorer for model A, particularly for observer 2, for whom the CITL was -1.67, calibration slope was 0.52, O/E ratio was 0.93, and Brier score was 0.09, indicating systematic overprediction in that combination of model and observer.

## Discussion

We previously developed two logistic models to predict IDH status in lower-grade (CNS WHO 2–3) diffuse glioma. In the initial development (15), model A emerged as the most accurate, but model B performed nearly as well, not requiring knowledge of calcification status (e.g., where CT is unavailable). Considering these merits, both models were subjected to external validation. The models were newly applied to predict the integrated diagnosis of mGBM as defined by 2021 CNS WHO in a new population of consecutively recruited diffuse glioma from a different geographical region. The previous first-round validation (University College London Hospitals) results in CNS WHO grades 2–3 (model A: 89% sensitivity, 80% specificity, 82% accuracy, AUC 0.96; model B: 67% sensitivity, 90% specificity, 86% accuracy, AUC 0.94 (15)) were surpassed by the new results (University Hospitals Nottingham; 97%–99% sensitivity, 48%–91% specificity, 87%–97% accuracy, and 0.93–0.98 AUC across both models and observers).

For the expert rater, the use of model B produced a marginal gain with specificity increased from 87% to 91.3% and accuracy from 96.3% to 97.2%. For the trainee rater, the use of models A and B each produced improvements in diagnostic performance. This increased the sensitivity for identification of IDH^wt^ mGBM from 87.1% to 97.6% (model A) and to 96.5% (model B). Model use improved the trainee’s accuracy from 84.3% to 87% (model A) and to 91.7% (model B). This newly achieved correct classification of non-contrast-enhancing mGBM supports a role for the tool in identifying atypical disease. For the trainee rater, model use increased NPV from 60.7% to 84.6% with model A and 85.0% with model B, with corresponding increases in F1 score from 0.90 to 0.92 and 0.95. Model B maintained a high PPV of 93.2%, indicating a favorable balance between sensitivity and positive predictive performance, although predictive values should be interpreted in the context of the high proportion of mGBM in this cohort. Calibration of model A was more observer-dependent, with less favorable calibration for observer 2 (trainee). On the other hand, model B demonstrated more consistent calibration across observers. The absence of CT for calcium assessment may have also contributed to the greater observer-dependent variation in the calibration of model A. Calibration estimates should, nevertheless, be interpreted in the context of the cohort composition, which included only 23 non-mGBM cases.

The new population was rich in CNS WHO grade 4 (by microscopy) tumors, which is explained by the consecutive enrolment strategy and aligns to glioma public datasets. Few (*n* = 2) CNS WHO grades 2–3 IDH^wt^ mGBM were identified in this study despite reviewing neuropathologist narrative data in addition to molecular results for all cases, where this group may represent up to 30% of all GBM (2). This discrepancy could be attributable to reduced emphasis on microscopy following the cIMPACT-NOW 3 and CNS WHO 2021 definitions of mGBM (1, 5). An influence from health service factors on the time to diagnosis also appears possible (18).

Other algorithms exist for glioma genotyping—for example, Kang et al. (2024) proposed the inclusion of the T2-FLAIR mismatch sign and tumor border definition (19); however, both of these features were excluded in our original model development because of limited (kappa <0.6) interobserver agreement contrary to their promising performance in other studies. Furthermore, we excluded perfusion imaging, which may not be universally available and may be variable for regional methods (20, 21).

Several studies highlighted the potential of machine learning methods for diffuse glioma genotyping, commonly targeted at IDH status prediction—for example, van der Voort et al. achieved high accuracies with a deep learning algorithm using anatomical imaging, without ADC or age for IDH and 1p19q genotyping (22). In the recent meta-analysis of machine learning radiomics approaches for IDH genotyping in gliomas by Chen et al., the pooled AUC values approached 0.90 (23). Computational radiomics approaches are attractive from a workflow perspective but require translational research to enter practice. Their clinical introduction is also restricted by the individuality of pipelines (24). Our method differs from this as an immediate option to support clinical MRI reporting.

Interestingly, many radiomics studies report high diagnostic accuracies for diffuse glioma genotyping but lack direct comparisons against human reasoning. Here the diagnostic value of computational tools may be overestimated where a radiologist opinion would perform well but is omitted. This could particularly be the case for datasets including high proportions of “classic” GBM and IDH^mut/^1p19q^intact^ low-grade astrocytomas (25).

In our current study, the interobserver agreement for morphological features was substantial to near perfect in several domains, similar to the prior work (15). However, no CT imaging was available for review, and the calcification assessments differed substantially between expert and trainee (kappa -0.016). GBM commonly exhibits petechial hemorrhages, whereas few 1p19q codeleted tumors featured in this study (26).

The assessment for cysts showed agreement (kappa 0.84), consistent with previous research (27, 28), despite the need to distinguish these from necrotic GBM regions. Interobserver agreement was confirmed for tumor location (kappa 0.90) and contrast-enhancement pattern (weighted kappa 0.82). The lower kappa result for the solid-patchy enhancement category is noted with a wide confidence interval (0.31–0.82) for this small group. Extensive literature is published on associations of IDH status and tumor location, including a known predilection of IDH^wt^ tumors for the temporal lobe and the thalamus (14, 29). For tumors overlapping multiple lobes, a choice for a single location was required for application of the model.

The interobserver agreement for regional rADC_mean_ measurements (ICC 0.79), despite the high proportion of necrotic mGBM in the study, suggests adequate reproducibility even in the presence of tumor heterogeneity. In a recent meta-analysis, ADC appeared to be a valuable biomarker of IDH status across diffuse glioma CNS WHO grades 2–4 (12). Regarding the ADC method, we recently observed non-inferiority of the regional rADC_mean_ method over volumetric ADC measurements using manual segmentation and deep learning automation (30). While this partial tumor measurement is counterintuitive, it appears sufficiently representative (11). Our current study included few molecular glioblastomas with an explicit CNS WHO 2–3 histomorphology description (*n* = 2) by the neuropathologist. Disregarding microscopy, in IDH^wt^ mGBM with atypical enhancement patterns, the use of the logistic calculation markedly improved the diagnostic accuracy of the trainee observer (from 12.5% up to 100%). This could be valuable for the identification of early aggressive disease but remains exploratory due to the small number (*n* = 8) in this group.

As a further limitation, the total case number of this validation (*n* = 108) is not large, and only two individuals conducted the MRI assessments (with six different observers previously contributing to model development). Both studies highlight the role of a simple logistic paradigm to identify atypical mGBM, although their proportion was small in the current study due to nonselective enrollment. Our framework predicts mGBM but is not focused on assessing 1p19q status. Neuroradiologist performance may have been enhanced by the knowledge of patient age. Model B appears preferable from the current study data, whereas model A performed minimally better for trainee mGBM identification, but with low specificity. In clinical practice, CT is often available to define calcification status.

## Conclusion

Our findings point toward the ability of a logistic paradigm to predict mGBM as defined in CNS WHO 2021, including for the small number of radiologically atypical tumors in the study. The tool enhanced accuracy, specifically the sensitivity for the identification of a lethal neoplasm, for a trainee rater. In an unselected diffuse glioma population, the genotyping performance of an experienced neuroradiologist appeared similar to that reported for radiomics approaches. Further validation is warranted, particularly in mGBM lacking rim enhancement.

## Data Availability

The data analyzed in this study is subject to the following licenses/restrictions: Available on request. Requests to access these datasets should be directed to Stefanie.Thust@nottingham.ac.uk.
