# Supplementary material for: A logistic paradigm to predict molecular glioblastoma on clinical MRI
Source: Front Oncol. 2026 Sep 28;16:1884886. doi: 10.3389/fonc.2026.1884886 (PMC13654790; doi:10.3389/fonc.2026.1884886)
Supplement: Supplementary file 1 [file Table1.docx]

# Supplementary Data

| **Comparison** | **System 1 AUC** | **System 2 AUC** | **DeLong Test** | **Holm-adjusted p-value** |
| --- | --- | --- | --- | --- |
| Observer 1 & Model B vs Observer 1 & Model A | 0.96 | 0.98 | -0.78 | 0.43 |
| Observer 1 & Model B vs Observer 2 & Model B | 0.96 | 0.94 | 2.15 | 0.16 |
| Observer 1 & Model B vs Observer 2 & Model A | 0.96 | 0.93 | 2.38 | 0.10 |
| Observer 1 & Model A vs Observer 2 & Model B | 0.98 | 0.94 | 1.68 | 0.25 |
| Observer 1 & Model A vs Observer 2 & Model A | 0.98 | 0.93 | 2.09 | 0.16 |
| Observer 2 & Model B vs Observer 2 & Model A | 0.94 | 0.93 | 1.72 | 0.25 |

**Supplementary Table 1** – Table represents paired DeLong comparisons among the four systems that generated continuous model probabilities. Differences are calculated as the first-listed system minus the second-listed system. Holm-adjusted p-values account for multiple comparisons within each test family. Comparisons involving simultaneous changes in both observer and model are exploratory, because these two effects cannot be separated.

| **Comparison** | **System 1 Accuracy** | **System 2 Accuracy** | **McNemar Statistic** | **Holm-adjusted p-value** |
| --- | --- | --- | --- | --- |
| Observer 1 vs Observer 1 & Model B | 0.96 | 0.97 | 2 | 1.00 |
| Observer 1 vs Observer 1 & Model A | 0.96 | 0.95 | 2 | 1.00 |
| Observer 1 vs Observer 2 | 0.96 | 0.84 | 0 | <0.01 |
| Observer 1 vs Observer 2 & Model B | 0.96 | 0.92 | 1 | 0.88 |
| Observer 1 vs Observer 2 & Model A | 0.96 | 0.87 | 0 | 0.02 |
| Observer 1 & Model B vs Observer 1 & Model A | 0.97 | 0.95 | 0 | 1.00 |
| Observer 1 & Model B vs Observer 2 | 0.97 | 0.84 | 1 | 0.01 |
| Observer 1 & Model B vs Observer 2 & Model B | 0.97 | 0.92 | 0 | 0.28 |
| Observer 1 & Model B vs Observer 2 & Model A | 0.97 | 0.87 | 1 | 0.04 |
| Observer 1 & Model A vs Observer 2 | 0.95 | 0.84 | 1 | 0.02 |
| Observer 1 & Model A vs Observer 2 & Model B | 0.95 | 0.92 | 0 | 0.88 |
| Observer 1 & Model A vs Observer 2 & Model A | 0.95 | 0.87 | 1 | 0.12 |
| Observer 2 vs Observer 2 & Model B | 0.84 | 0.92 | 3 | 0.46 |
| Observer 2 vs Observer 2 & Model A | 0.84 | 0.87 | 7 | 1.00 |
| Observer 2 & Model B vs Observer 2 & Model A | 0.92 | 0.87 | 1 | 0.88 |

**Supplementary Table 2** – Table presents two-sided exact McNemar comparisons of paired classification accuracy among all six observer–method systems. Differences are calculated as the first-listed system minus the second-listed system. Holm-adjusted p-values account for multiple comparisons within each test family. Comparisons involving simultaneous changes in both observer and model are exploratory, because these two effects cannot be separated.

| **Observer** | **Expected events** | **Calibration Intercept (95% CI)** | **Calibration Slope (95% CI)** | **Calibration-in-the-large (95% CI)** | **O/E Ratio (95% CI)** | **Brier Score (95% CI)** |
| --- | --- | --- | --- | --- | --- | --- |
| Observer 1 & Model A | 85.65 | 0.02 (-1.03 - 1.07) | 0.73 (0.39 - 1.06) | -0.26 (-1.47 - 0.96) | 0.99 (0.94 - 1.03) | 0.04 (0.01 - 0.07) |
| Observer 1 & Model B | 82.37 | 0.45 (-0.50 - 1.40) | 1.13 (0.62 - 1.64) | 0.49 (-0.38 - 1.36) | 1.03 (0.99 - 1.08) | 0.04 (0.01 - 0.06) |
| Observer 2 & Model A | 91.81 | -0.52 (-1.45 - 0.41) | 0.52 (0.30 - 0.75) | -1.67 (-2.58 - -0.76) | 0.93 (0.86 - 0.98) | 0.09 (0.05 - 0.13) |
| Observer 2 & Model B | 85.40 | 0.04 (-0.75 - 0.83) | 0.88 (0.50 - 1.25) | -0.06 (-0.83 - 0.70) | 1.00 (0.94 - 1.05) | 0.06 (0.03 - 0.10) |

**Supplementary Table 3** – Calibration measures for the Model A (calcium) and Model B (cyst), evaluated separately for Observers 1 and 2 in 108 patients. Results are presented with 95% confidence intervals. Ideal calibration corresponds to a calibration intercept and CITL of 0, a calibration slope of 1, and an O/E ratio of 1; lower Brier scores indicate greater overall accuracy of the predicted probabilities. Confidence intervals were estimated using 2,000 patient-level bootstrap resamples for O/E and Brier intervals, with Wald confidence intervals for intercept, slope and CITL. CITL, calibration-in-the-large; O/E, observed-to-expected ratio.

**Supplementary Figure 1** – Logistic recalibration curves for the cyst model (B) and calcium model (A), evaluated separately for observers 1 and 2 in 108 patients. The dashed diagonal line represents perfect calibration, the solid blue line represents the fitted logistic recalibration curve, and the shaded region represents its patient-level bootstrap 95% confidence interval based on 2,000 resamples. Red circles show the observed event proportion within quantile groups plotted against the corresponding mean predicted probability; circle size reflects the number of patients in each group. Rug marks along the x-axis show the distribution of individual predicted probabilities. CITL, calibration-in-the-large; O/E, observed-to-expected ratio.
